# Biomechanics of open-globe injury: a review

**DOI:** 10.1186/s12938-023-01117-8

**Published:** 2023-05-25

**Authors:** Songtao Wang, Fuqiang Li, Siyan Jin, Yu Zhang, Ning Yang, Jinsong Zhao

**Affiliations:** Department of Ophthalmology, The Second Hospital of Jilin University, No. 4026, Yatai Street, Nanguan District, Changchun, Jilin China

**Keywords:** Open-globe injury, Biomechanics, Force, Intraocular pressure, Stress

## Abstract

Open-globe injury is a common cause of blindness clinically caused by blunt trauma, sharp injury, or shock waves, characterised by rupture of the cornea or sclera and exposure of eye contents to the environment. It causes catastrophic damage to the globe, resulting in severe visual impairment and psychological trauma to the patient. Depending on the structure of the globe, the biomechanics causing ocular rupture can vary, and trauma to different parts of the globe can cause varying degrees of eye injury. The weak parts or parts of the eyeball in contact with foreign bodies rupture when biomechanics, such as external force, unit area impact energy, corneoscleral stress, and intraocular pressure exceed a certain value. Studying the biomechanics of open-globe injury and its influencing factors can provide a reference for eye-contact operations and the design of eye-protection devices. This review summarises the biomechanics of open-globe injury and the relevant factors.

## Background

Ocular trauma is the leading cause of monocular blindness worldwide [1], with more than 55 million cases reported each year, of which 1.6 million result in loss of vision. Approximately 200,000 ocular trauma cases are open-globe injury (OGI) [2]. OGI include penetrating ocular injuries, perforating ocular injuries, ocular rupture, and intraocular foreign bodies [3] and can cause severe damage to intraocular tissue and vision. In China, OGI was most commonly found in people aged 47.8 ± 14.1, with more male patients than female [4, 5]. In the elderly, falls are the main cause of open-globe injuries [6, 7]. In young people, over one-third of all OGI were found to be work related, and 89.1%, of these patients had not worn adequate eye protection [8]. In addition, children are at high risk for eye injuries. According to previous reports, eye injuries in children occur from 6 months to 14.9 years of age and are more common in boys [9, 10]. The family home is the most common scene of these accidents, and most injuries are caused by sharp tools such as knives. Worldwide, penetrating trauma is the most common form of OGI in children, accounting for 48.4–83% of all OGI, followed by eyeball rupture at 9.9–34% [11]. Research has shown that the vast majority of eye injuries, whether in adults or children, are preventable [12–14]. Therefore, particular attention should be paid to the prevention of eye injuries in occupations prone to ocular trauma such as farming, and in children [8, 15, 16]. Studying the biomechanics of OGI and its influencing factors can provide information useful for the evaluation of OGI risk and for prevention.

## Search strategy

A systematic literature search was conducted in January 2023. We searched PuMed, Web of Science (WOS) and Chinese National Knowledge Infrastructure (CNKI) with a hierarchical search strategy by using the following Medical Subject Heading (MeSH) terms and text words: "eye injuries, penetrating", "globe rupture", "ocular rupture", "open globe injuries", "stress, mechanical", "biomechanical phenomena", "physical stimulation", "intraocular pressure" and "rupture/etiology", without limit of date, language and article type restriction. Next, we delete duplicate references in the search results and search for all references to obtain the full text. Finally, two authors screened the literatures that meets the requirements by reading the article title and abstract. The inclusion criteria of this review including: (1) the full text of the literatures can be obtained; (2) the literatures should be related to open-globe injuries; and (3) the literatures should focus on the biomechanical parameters or numerical simulations of eye injuries.

## Ocular structure

The eyeball is composed of a wall and intraocular tissue. The eyeball wall comprises the cornea, sclera, choroid, and retina. The intraocular tissues include the aqueous humour, iris, crystalline lens, zonular fibres, and vitreum. Compared with the choroid and retina, the cornea and sclera play a more important role in maintaining eyeball wall stability [17, 18]. Therefore, in studies of OGI, the eyeball wall is described as corneoscleral. The thickest parts of the eyeball wall are located at the optic nerve outlet and the corneal limbus, with a thickness of approximately 1000 μm. Conversely, the thinnest portion is located at the attachment of the rectus muscles, having a thickness of approximately 300 μm [19, 20]. During blunt trauma, the weaker parts of the eyeball such as the attachment of the extraocular muscles, the corneoscleral limbus, and the equatorial part of the eyeball, can rupture due to rapidly increasing intraocular pressure (IOP). Penetrating injuries to the eyeball are usually caused by sharp foreign bodies piercing or puncturing the eyeball wall, and the wound is usually located at the point of contact between the foreign body and the eyeball. These wounds can be divided into penetrating injuries of the cornea, corneosclera, and sclera, and usually involve greater damage to the anterior segment [21]. In addition to the influence of eyeball structure, the risk and location of OGI are also related to certain biomechanical effects.

## Biomechanics

### Force and unit area impact energy

#### Threshold force to penetrate the eyeball

**Fig. 1:**
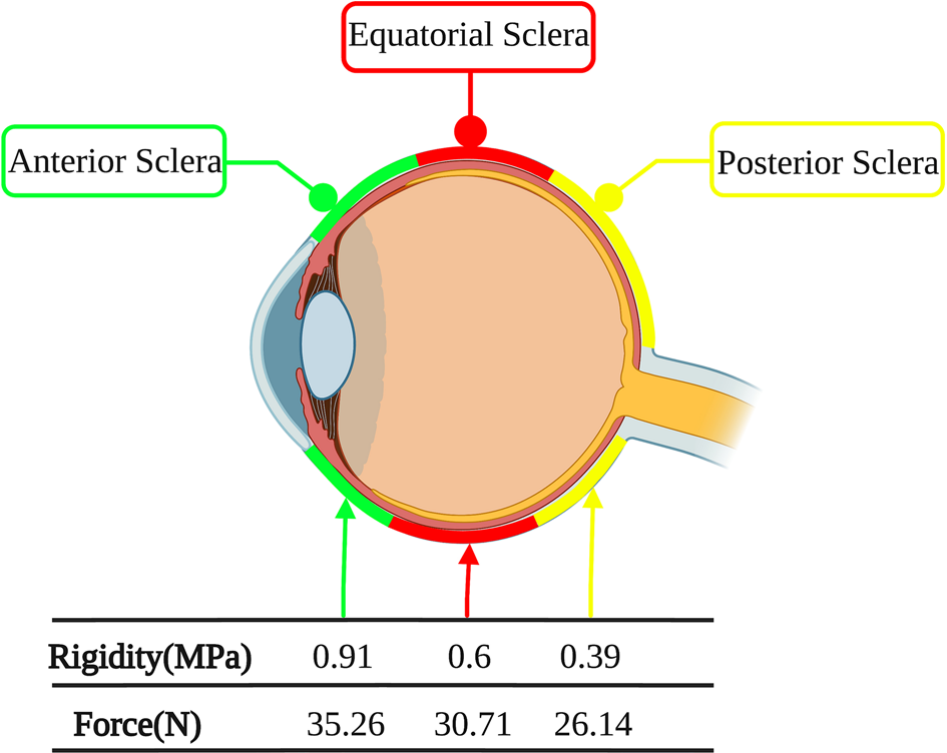
The rigidity and penetrating force of different parts of Scleral

Although the cornea and sclera are both part of the eyeball wall, the forces required to penetrate them differ. Lovald [21] applied pressure to the central cornea of 36 human cadaver eyes at a speed of 1 or 5 mm/s using flat-headed probes of different diameters to measure the force required to penetrate the cornea. The force required positively correlated with the probe diameter. The force was 30.5 ± 5.5 N using a 1-mm-diameter probe, 40.5 ± 8.3 N using 1.5 mm, and 58.2 ± 14.5 N using 2 mm. However, the rupture of eyeball is independent of the speed of the probe. The forces of penetration through different regions of the sclera differ because of different wall rigidities. Rigidity is the ability of a material to resist deformation; the greater the rigidity, the greater the ability to resist deformations caused by external forces. Fan [22] used a 1-mm flat-headed probe to exert pressure on porcine scleras at a speed of 1 mm/min. The force required to penetrate the sclera differed between areas, being 35.26 ± 4.72 N at the anterior sclera, 30.71 ± 3.91 N at the equatorial sclera, and 26.14 ± 3.28 N at the posterior sclera. This result is consistent with the variations in the rigidity of the different parts of the sclera. For example, the rigidity of the anterior sclera was greatest at 0.91 ± 0.21 MPa, that of the equatorial sclera less at 0.6 ± 0.16 MPa, and that of the posterior sclera least at 0.39 ± 0.13 MPa (Fig. 1). In addition to stiffness, the force required for a foreign object to penetrate the eyeball wall is closely related to its sharpness and inversely to its diameter. When the contact area between the foreign object and eyeball decreases, the force required to pierce the eyeball wall also decreases. Compared with Fan’s results, Park [23] significantly reduced the minimum sclera-penetrating force by replacing the flat-head probe with a slanted-head probe; the force required for puncturing the porcine anterior sclera using a 1.1-mm-diameter scleral puncture needle was found to be only 1.25 N. In human scleras, the penetration force of the same diameter of needle was significantly less than in the porcine eye. Matthews [24] used an 18G oblique needle with a diameter of approximately 1.21 mm to pierce each area of the human eye in vitro. The forces required were 0.75 N at the limbus, 0.95 N at the front of the sclera, 0.7 N at the equator of the sclera, and 1 N at the posterior pole of the sclera. The reason for the lower forces may be that in the human eye, the thickness of the sclera is less, being approximately half that of its porcine counterpart [25]. The data in Table 1 reflect the force required to puncture the sclera at a distance of 3 mm from the corneal margin using puncture needles of various diameters. From this it can be seen that foreign objects with smaller diameters can penetrate the eyeball wall more effectively and require less force. However, the force measured using needles of the same diameter to pierce the same experimental eye fluctuate within a certain range. Park [23] used 27G and 30G puncture needles to pierce the porcine eye and measured greater forces than did Christensen [26]. The reasons for this may include age differences in the experimental animals, whether the experimental eye had been frozen, and human factors. For example, Park [23] used porcine eyes that were detached for 7 h but Christensen [26] used porcine eyes that were detached for less than 3 days. In addition, these investigators used regularly shaped puncture needles; however, the morphology of the wounds seen clinically in sharp-instrument open-eye injuries is mostly irregular. Therefore, further experiments are required to simulate realistic injury environments. Finally, the use of animal eyes is prevalent in the literature owing to the scarcity of human eye donors, which is another limitation. Nonetheless, we expect that the data in Table 1 can provide a reference for constructing eye-injury prediction models and conducting eye-injury simulation experiments.

**Table 1:** The force required for puncturing the sclera 3.5–4 mm from the limbus of cornea with different diameters

Study	Eyeball	Needle	Diameter	Force (N)
Park et al. [23]	Porcine	27G	0.40	1.30 ± 0.30
		30G	0.30	0.90 ± 0.30
		33G	0.20	0.75 ± 0.21
Christensen et al. [26]	Porcine	19G	1.10	1.25
		25G	0.50	0.89
		27G	0.40	0.91
		30G	0.30	0.45
		32G	0.23	0.44
Pulido et al. [27]	Human eye	27G	0.40	0.61
		30G	0.30	0.23
		31G	0,25	0.29
Matthews et al. [24]	Human eye	18G	1.20	0.95

#### Effect of force and unit area impact energy on the eyeball

In addition to penetrating injury to the eyeball, ocular rupture caused by blunt impact is also very common. Many patients suffer from ocular injury or even blindness owing to the impact of bombs, air gun projectiles, or metal objects [28]. Kennedy [29] impacted the FOCUS head mould (Facial and Ocular Countermeasure Safety head form) with a 4.5-mm BB projectile to determine the risk standard for ocular rupture. These researchers found that the force causing a 50% risk of ocular rupture was 107 N. In addition to force, the degree of eye injury was positively correlated with the energy of the impactors. Sponsel [30] impacted porcine eyeballs with polyethylene glycol-filled gelatin capsules ('paintballs') having 2–13.5 J (J) of energy and found that the pathological changes increased with increasing energy. Two joules of energy caused lens dislocation and choroidal detachment, 4 J caused anterior dislocation of the lens, 7 J caused the iris and ciliary body root to tear, and 10 J caused eyeball rupture. However, in addition to regularly shaped objects such as paintballs and BBs, clinicians encounter injuries due to many types of irregular impactors, including wooden sticks, fists, and blunt metal objects [31]. The geometries and masses of these impactors vary significantly. Therefore, the unit area impact energy (E/a) has been proposed as a predictor of eye injury. E/a is the ratio of the energy of the impactor to the impact area. Researchers believe that the E/a value can predict the degree of eye injury and tissue lesions more accurately than can the energy of the impactor alone and is the best predictor of eye injuries [32]. Research has shown that the E/a values of a 50% risk of corneal contusion, lens dislocation, hyphema, retinal injury, and ocular rupture are 1503 J/m^2^, 19,194 J/m^2^, 20,188 J/m^2^, 30,351 J/m^2^, and 23,771 J/m^2^, respectively, indicating that the degree of eye injury increases with an increase in E/a [30]. To measure the E/a value of ocular rupture, Kennedy [33] impacted porcine and human eyeballs with objects such as BBs, aluminium rods, foam, and baseballs. They found that human and porcine eyeballs had a 50% risk of rupture at impact kinetic E/a values of 35,519 J/m^2^ and 71,145 J/m^2^, respectively, indicating that the E/a required for porcine ocular rupture was approximately twice that of human eyes. The structural differences between porcine and human eyes resulted in differences in the parameters of eye trauma. Because of the scarcity of human eye donors, these differences should be considered when using porcine data to perform numerical simulation tests of eye injuries in humans. Marshall [34] impacted a porcine eye with metal and plastic marbles of different diameters, and calculated that the E/a threshold of corneal rupture was approximately 45,500 J/m^2^. As the best factor for predicting eye injury, we hope that the E/a value in Table 2 can provide a reference for clinicians to judge the risk of eye injury and help predict the risk of eye injury by consumer products.

**Table 2:** The energy required for causing different types of eye damage

Study	Method	Eyeball	Result	
Sponsel et al. [30]	Eyeball impacting	Porcine	Lens dislocation and choroidal detachment (2 J), anterior lens dislocation (4 J), iris and ciliary body root detachment (7 J), eyeball rupture (10 J)	
		Porcine	50% risk of eye injury: corneal contusion (1503 J/m^2^), lens dislocation (19,194 J/m^2^), hyphema (20,188 J/m^2^), retinal injury (30,351 J/m^2^), eye rupture (23,771 J/m^2^)	
Kennedy et al. [33]		Human eyeball	50% risk of injury	Ocular rupture (35,519 J/m^2^)
		Porcine		Ocular rupture (71,145 J/m^2^)
Marshall et al. [34]		Porcine	Corneal rupture (45,500 J/m^2^)	

### Rupture stress

Stress refers to the interaction forces between various parts of an object when deformed by an external force. In the absence of stress, the collagen fibres of the eyeball wall appear curled under an electron microscope [35]. When the eyeball is impacted by a blunt object, the IOP increases rapidly and the eyeball wall expands towards the equator or the front and rear poles. During this process, the curled collagen fibres gradually straighten and the stress on the eyeball wall increases. The rupture stress refers to the maximum stress that a material can withstand. If the stress exceeds this value, the material is destroyed. When the eyeball is injured, the stress gradually increases, reaches its ultimate limit, and rupture ensues. Table 3 lists the ultimate stresses acting on eyeball walls. Presently, in vitro simulation experiments on eye injuries mainly depend on eyeball models and animal eyes. Data on the ultimate stress of eyeball wall rupture can help experimenters select suitable model materials and design numerical eyeball models. These models will permit biomechanical eye experiments to be performed in simulation to obtain more realistic and reliable data.

**Table 3:** Rupture stress required for ocular rupture

Study	Method	Eyeball	Rupture stress (MPa)	
Bisplinghoff et al. [36]	Intraocular compression	Human eyeball	Corneal	13.89
Takahashi et al. [37]	Eyeball impact	Human eyeball model	Corneal	9.45
			Sclera	9.49
Stitzel et al. [38]	Eyeball impact	VT-WFU eyeball model	23	

### IOP

IOP refers to the pressure produced by the contents of the eyeball on the eyeball wall. Its stability depends on the balance between eyeball content and eyeball volume. The normal IOP is 10–21 mmHg. When the eyeball is impacted, its volume is rapidly compressed, leading to a sharp increase in the IOP. When the pressure exceeds a certain range, the eyeball ruptures. The most common rupture position in tests is close to the equator of the sclera because the impact position is usually the centre of the eyeball, and the eyeball, thus, expands towards the equator of the sclera and the posterior pole of the eye [39]. The IOP of ocular rupture is usually measured by intraocular compression. These data are shown in Table 4. It can be seen that the rupture IOP increases with the rate of increase of the IOP, indicating that the threshold IOP of rupture is not only related to the structure of the eyeball itself, but also to the force and speed of the impactor. The greater the force and speed, the faster the volume of the eyeball changes and the higher the IOP of the ocular rupture. Bispinghoff [40] applied 36.5 MPa/s of pressure to the inner part of the eyeball to simulate the severe impacts sustained by the eyeball during traffic accidents and movement. The IOP at ocular rupture did not change significantly with a sharp increase in the compression rate, indicating that the IOP at rupture only increased with an increase in the compression rate within a certain range. The IOP of ocular rupture can not only be used by clinicians to determine the safety and effectiveness of surgery, but can also be used to predict the risk of eyeball rupture or to verify a laboratory eyeball model. For example, when the rate of IOP rise is 0.02 ± 0.01 MPa/s, the risk function predicts that the eyeball has a 50% risk of rupture when the IOP is 0.35 MPa. When the rate of IOP rise is 2.77 ± 0.58 MPa/s, the eyeball has a 50% risk of rupture when the IOP is 0.90 MPa [39]. However, it should be noted that the pressure of eyeball rupture is related to species. Table 4 shows that the static and dynamic rupture pressures of the porcine eye are higher than those of the human eye, which should be borne in mind when conducting human-relevant experiments or clinical modelling.

**Table 4:** Ruptured IOP of eyeball

Study	Method	Eyeball	Pressurization rate (MPa/s)	Ruptured IOP (MPa)
Kennedy et al. [39]	Intraocular compression	Human eyeball	2.77 ± 0.58	0.91 ± 0.29
			0.02 ± 0.01	0.36 ± 0.20
		Porcine	2.77 ± 0.58	1.64 ± 0.32
			0.02 ± 0.01	1.00 ± 0.18
Bisplinghoff et al. [40]	Intraocular compression	Human eyeball	36.50 ± 15.35	0.97 ± 0.29
Burnstein et al. [41]	Intraocular compression	Human eyeball	0.006	0.46 ± 0.12
		porcine		0.53 ± 0.10

## Factors affecting the biomechanics of ocular rupture

### Impact location

Several types of clinical eye injuries can occur, both indirect and direct. When the eyeball is directly injured, the location determines whether the impacting object causes an ocular rupture. Research has shown that if an impact on the central cornea causes rupture, the same impact on a deviated position may not [21]. The explanation may be that when the central cornea is impacted, the eyeball can only expand towards the equator and posterior pole of the eyeball, and the eye axis is extremely compressed. However, when the impact site deviates from the central cornea, the eyeball expands towards the equator and both anterior and posterior poles. The degree of eyeball compression is therefore low, and the possibility of eyeball rupture is reduced under these conditions.

Indirect ocular injury is caused by the shock wave of a craniocerebral trauma being transmitted to the eyeball. Research has shown that when the head is impacted, the frontal bone transmits most of the kinetic energy to the eyeball, accounting for 86.8% of the total kinetic energy of the impactor, followed by the eyebrow (73.3%), temporal bone (62.3%), and zygomatic bones (40%) [42]. The energy delivered to the eyeball by the frontal bone is more than twice that of the zygomatic bone, suggesting that the possibility of ocular rupture is greater when the frontal bone is the impact site in head trauma [43].

### Eye structure

Reportedly, with each year of age of the eye donor, the force threshold for corneal penetration decreases by 0.42 N [21]. This may be caused by a gradual hardening of the cornea, reductions in compliance, and a weakening of mechanical-stress buffering effects with advancing age. However, corneal stress-bearing capacity is also related to the eyeball diameter. Each 1-mm increase in the diameter of the eyeball reduces the force threshold for corneal rupture by 3.39 N [21]. This may be because the larger the diameter of the cornea, the smaller the thickness of the central and peripheral areas [44]. In addition to physiological and structural changes, pathological changes such as myopia and corneal refractive surgery affect the stress-bearing capacity of the eye. The axis of the eye is a hypothetical line from the centre of the cornea to the optic nerve and fovea of the retina. The length of the eye axis gradually increases as myopia progresses. Takahashi [37] studied the degree of damage to eyeballs with different axial lengths caused by BBs of different speeds and found that impact at a speed of 60 or 75 m/s on the eyeball may cause corneal tears. The longer the axial length, the greater is the deformability of the eyeball and thus the volume compression, suggesting that the risk of eyeball rupture is positively correlated with the severity of myopia. Uchio [45] studied the biomechanics of eyeball rupture caused by the high-speed impact of airbags after corneal refractive surgery and found that, compared with normal eyes, the bearing capacity of the operated eye was significantly reduced, and the risk of intraocular structural damage caused by the blunt contusion of an airbag was greater than that of normal eyes. The reason for the decrease in corneal endurance after corneal refractive surgery may be that eyeball strength after radiation keratotomy is significantly reduced, and the force that causes rupture of the operated eye is only 50–70% of that of a normal eye [46]. Finally, habitual rubbing of the eyes is a risk factor for keratoconus, a pathological state of the cornea [47]. Reportedly, long-term eye rubbing can cause the cornea to become thinner and reduce its stiffness, which may ultimately lead to a decrease in the ability of the cornea to resist external forces, resulting smaller forces to cause corneal rupture [48].

### Eye appendages

The accessory organs of the eye, including the eyelid, conjunctiva, lacrimal apparatus, extraocular muscles, and orbit, support and protect the eye. When the eyeball is stimulated by trauma or a foreign body, the orbicularis oculi muscle contracts instantly to close the eyelids and prevent injury [49]. Research has shown that the most common site of periocular tissue tears in open-eye trauma is the eyelid, and more commonly the upper eyelid [50]. Of eye trauma cases, 44% were associated with eyelid injuries [51]. In addition, six extraocular muscles are attached around the eyeball: the upper, lower, inner, and outer rectus muscles, as well as the upper and lower oblique muscles. The eyeball rotates to different directions depending on the contraction and relaxation of different extraocular muscles. The eyeball wall is thinnest at the attachments of these muscles. Some researchers have studied the protective effect of the extraocular muscles against eyeball injury and found no difference in the degree of injury when the extraocular muscles are removed or retained [52], indicating that the protective effect of extraocular muscles in eye injury is small. The orbit is the supporting structure of an eyeball. It has been reported that the smaller the horizontal diameter of the orbital opening, the more the eyebrow arch protrudes, the less the eye protrudes, and the more the eye is protected [53], indicating that when the eye is traumatized, the possibility of an object directly impacting the eyeball to cause eyeball rupture is reduced. However, research has shown that the larger the scope of the orbit around the eyeball, the greater is its ability to inhibit the propagation of shock waves to the eye, leading to a greater protective effect [54].

### Injury type

The causes of ocular trauma can be various. Compared to eye-contact injury type, the non-contact eye injury is also common, such as blast wave. Patients with ocular trauma caused by blast waves are common in clinical practice [55]. Military operations and holiday celebrations are common injurious scenes [56, 57]. Blast wave is characteristic of high conduction velocity and pressure-rise speed. The IOP rapidly increases dramatically and may exceed the threshold of the IOP of ocular rupture when the eye contacts the blast wave. Alireza [58] used the VT-WFU eyeball model and different weights of IED (improvised explosive devices) to simulate the damage of blast wave. Results shown that the higher the IED, the faster the increase in IOP. IOP elevation of 2900 and 2700 mmHg were observed after the blast for the IEDS weight of 2 kg and a victim distance of 2 m in front and side blasts, respectively. The rate of IOP increase is the fastest when the weight of the IED is 2 kg and 2 m away from the eyeball. And the stress of explosives in all directions is concentrated on the temporal sclera behind the equator of the eyeball. The reason may be that the temporal sclera lacks protection from the orbital bone compared to the nasal side. In addition, compared to IED placed off the ground, the blast waves caused by IED placed on the ground cause greater damage to the eyeball [59]. Because the blast wave generated by the IED on the ground will rebound through the ground [60], the rebound and enhanced shock wave will further increase the stress and strain of the sclera and increase the risk of eyeball rupture.

In addition to ocular rupture caused by ocular trauma, iatrogenic ocular rupture has also been reported, which should be prevented [61]. For example, the anesthetic is accidentally injected into the eyeball during retrobulbar anesthesia, resulting in ocular rupture caused by rapidly increased IOP. Bullock [62] simulated this condition by injecting physiological saline into rabbits’ eye. The results showed that the rupture spot of the rabbit eyes was located at the corneal limbus, sclera, and posterior pole. Besides, the anterior segment was normal in four of five of ruptured rabbit eyes in accordance with previous clinical cases. The required IOP for ocular rupture caused by accidental intraocular injection is between 2800 and 6400 mmHg, and it is easier to achieve the level using a 3 ml syringe than a 10 ml [63]. Therefore, it is necessary to measure the IOP for ocular rupture, which can also help prevent iatrogenic ocular rupture, such as using relatively large volume syringe, massaging the eyes during anesthesia, and manually evaluating IOP.

## Innovation points

Rupture of the eyeball is one of the most critical diseases in ophthalmology and often occurs in high-activity scenarios such as sports, military exercises, and car accidents. Rupture of the eyeball is a common cause of visual impairment, causing a serious economic burden to society and patients, as well as serious psychological problems for the patient. Biomechanics is an important aspect of open-eye injury, and many researchers have studied some of the eye’s biomechanical parameters; however, no relevant literature review has appeared to date. Therefore, this article considers the biomechanics of eye rupture as a starting point to analyse and summarise a series of parameters required to cause open-eye trauma, such as force, standardised energy, intraocular pressure, and stress, and analyses the factors that affect these parameters, such as the site of eye trauma, eye structure, eye accessory organs, iatrogenic parameters, and eye diseases.

## Summary

Quantifying the biomechanical conditions of eyeball rupture has significant clinical and societal value. First, these parameters can provide intuitive data for clinical physicians and can be used to: (1) analyse the relationship between the biomechanical conditions of eye injury and the degree of injury to provide a reference for formulating standards for eye injury; (2) provide a reference for the force exerted by instruments on the ocular surface during eye surgery (such as external scleral pressure and scleral puncture) to avoid iatrogenic damage. Second, common toy guns, slingshots, and other consumer products are prone to causing eye damage owing to excessive firing energy. The biomechanical parameters of eye rupture can also provide a reference for designing the diameter, weight, initial velocity, and energy of the projectiles fired by toy guns, as well as for designing eye protection. However, owing to the scarcity of donated human eyeballs and the limiting factors of the age of the eyeball donor, the diameter of the eyeball, the thickness of the eyeball wall, a history of eye surgery, and the experimental eye having been frozen, shortcomings in the study of human eye-related parameters remain. It is hoped that in the future, researchers building on the present foundation will be able to continuously improve the experimental parameters, measure more accurate and reliable data, and provide high-quality and valuable guidelines for preventing eye-rupture injuries.

## Data Availability

Not applicable.

## Abbreviations

OGI: Open-globe injury
IOP: Intraocular pressure
E/a: Unit area impact energy
FOCUS: Facial and Ocular CountermeasUre Safety headform
VT-WFU: Virginia Tech-Wake Forest University
